# Evaluation of the Impact of a Coordinated Intervention Model for Complex Older Patients with Dementia

**DOI:** 10.5334/ijic.10026

**Published:** 2026-05-26

**Authors:** Matilde Barneto-Soto, Núria Molist-Brunet, Emma Puigoriol-Juvanteny, Anna Ribera Nadal, Rosa María Torres Allepuz, Joan Espaulella-Panicot

**Affiliations:** 1 Territorial Service of Geriatrics and Palliative Care from Osona and Ripollès, Fundació Hospital Universitari de la Santa Creu-Consorci Hospitalari Vic, Hospital de Campdevànol, Catalonia, Spain; 2 Central Catalonia Chronicity Research Group (C3RG), Institut de Recerca i Innovació en Ciències de la Vida i de la Salut de la Catalunya Central (IRIS-CC), 08500 Vic, Catalonia, Spain; 3 Parc Sanitari Pere Virgili, Barcelona, Catalunya, Spain; 4 Universitat de Vic-Universitat Central de Catalunya (UVic-UCC), Spain; 5 RE-FIT Barcelona Research Group, Vall d’Hebron Institute of Research (VHIR), Catalunya, Spain; 6 Universitat Autònoma de Barcelona, Barcelona, Catalunya, Spain; 7 Consorci Hospitalari Vic, Catalonia, Spain; 8 Multidisciplinary Inflamation Research Group (MIRG), Institut de Recerca i Innovacióe n, Ciències de la Vida i de la Salut a la Catalunya Central (IRIS-CC), 08500 Vic, Catalonia, Spain

**Keywords:** integrated care, older, dementia, multimorbidity, frailty, person centred care, atenció integrada, gent gran, demència, multimorbiditat, fragilitat, atenció centrada en la persona

## Abstract

**Introduction:**

Person centred care planning and models of care coordination across levels of care reduce fragmentation and unnecessary resource use. Our objectives were to assess the impact of implementing a coordinated care model between intermediate care and primary care, as determined by the identification of complex patients, and recording their individualized care plans in the shared medical record and to assess its effectiveness in relation to the use of health resources.

**Methods:**

A pre-post quasi-experimental study of 160 complex patients with dementia who were discharged from intermediate care between April 2022 and July 2024.

**Results:**

A 31.4% (from 63 to 106 patients) increase in the identification of complex patients with dementia and a 23.6% (from 19 to 44 patients) increase in the development of care plans were found at 3 months of discharge. Of the patients who had visited emergency department in the six months preceding hospitalisation, 54.4% (n = 31) did not visit it in the six months post-discharge (p = 0.026).

**Conclusion:**

Our coordinated care model may have contributed to improved identification of patients with dementia and complex conditions, the development of individualized care plans, and reduced emergency department use.

## Abstract (ca)

**Introducció:**

La planificació de l’atenció centrada en la persona i els models de coordinació assistencial entre nivells assistencials redueixen la fragmentació i l’ús innecessari de recursos. El nostre objectiu va ser avaluar l’impacte de la implementació d’un model d’atenció coordinada entre l’atenció intermèdia i l’atenció primària, determinat per la identificació de pacients complexos i el registre dels seus plans d’atenció individualitzats a la història clínica compartida, així com avaluar-ne l’efectivitat en relació amb l’ús de recursos sanitaris.

**Mètodes:**

Estudi quasi experimental pre-post de 160 pacients amb complexitat i demència donats d’alta de l’atenció intermèdia entre abril de 2022 i juliol de 2024.

**Resultats:**

Es va observar un augment del 31,4% (de 63 a 106 pacients) en la identificació de pacients complexos amb demència i un increment del 23,6% (de 19 a 44 pacients) en l’elaboració de plans d’atenció als 3 mesos de l’alta. Dels pacients que havien visitat el servei d’urgències en els sis mesos previs a l’hospitalització, el 54,4% (n = 31) no el van visitar en els sis mesos posteriors a l’alta (p = 0,026).

**Conclusió:**

El nostre model d’atenció coordinada pot haver contribuït a millorar la identificació de pacients amb demència i condicions complexes, el desenvolupament de plans d’atenció individualitzats i a reduir l’ús dels serveis d’urgències.

## Background

Fragmented care within the healthcare system and hospital care focused on single diseases do not adequately respond to the needs of older patients with multimorbidity (two or more chronic diseases) and/or frailty [1]. This situation results in growing demand for healthcare resources and increased consumption of drugs, which have a significant impact on both costs and patient quality of life [1,2,3,4]. Faced with this situation, healthcare systems must evolve by developing models that provide personalized, integrated care by multidisciplinary teams, co-ordinated across different care settings and are cost-effective. Person centred care planning should be a key element of the care planning process as it allows patients to actively participate in shared decision making (SDM) with the members of the care team in developing an individualised care plan (ICP) that considers its values and its main care goal (prolonging survival, maintaining function or prioritising symptom control) [5,6,7]. The paradigm shift towards a model in which care is planned based on the patient’s needs, their values and preferences can help to decrease the fragmentation of care, reduce unnecessary use of hospital and emergency services and improve the patient’s experience [3,8].

In order to support care management for this segment of the population, Catalonia’s Department of Health implemented the Chronic Care Programme. Two patient profiles were identified: i) patients with chronic conditions and complex care needs (CCP), based on the presence of a combination of clinical complexity, and/or complex social factors, and/or complex health system factors [9] and; ii) patients with advanced chronic diseases and limited life expectancy (ACP). The NECPAL CCOMS-ICO© version 4.0 tool is used to assist in early identification of these patients [10,11]. To respond to the needs of CCP and ACP patients, a four-stage person centred care model has been developed, consisting of: a) identification of the patient’s profile (mild frailty, moderate frailty or CCP, and advanced frailty or ACP); b) a situational diagnosis [12], which requires a multidimensional assessment and assessment of needs; c) putting together an ICP within a SDM framework involving the practitioners and the patient [13], and; d) the sharing of information between the practitioners and care settings involved in the patient’s care [9]. In the Catalan healthcare system, primary care (PC) teams record patients identified as needing CCP or having ACP and their ICPs in the shared care record (SCR), which allows this information to be available in all care settings.

Persons with dementia have more comorbidities and approximately twice as many hospital stays per year compared to older adults without dementia, resulting in significantly higher healthcare costs. In Catalonia, dementia is associated with a 44.1% increase in total healthcare costs, with an attributable additional expenditure of €1,311.7 per person per year, mainly related to hospitalisations, ED use and IC [14]. These hospitalisations are associated with poorer health outcomes such as cognitive and functional decline, delirium, falls, institutionalisation, and mortality [15,16,17,18]. Beyond clinical and economic outcomes, it is important to emphasise the negative experiences of people with dementia and, in particular, their carers in the ED, emphasising communication difficulties, stress associated with the healthcare environment and the perception of potentially avoidable visits and readmissions [19]. It is therefore clear that since patients with dementia are usually older, with chronic conditions and complex care needs, require an integrated and person centred care model [20].

The objectives of this study were to: i) assess the implementation of a coordinated care model between intermediate care (IC) and PC, as determined by an increase in record-keeping by the PC teams in the SCR, the identification of patients with dementia and complex and advanced chronic conditions, and the development of ICPs for patients with dementia and complex or advanced chronic conditions, and; ii) assess the effectiveness of the model in relation to use of healthcare resources.

## Method

### Study Design and participants

A pre-post quasi-experimental study without control group was conducted to assess the implementation and effectiveness of a coordinated care model between IC and PC teams.

In this study, 160 patients with a diagnosis of dementia who were admitted consecutively to the Psychogeriatric Unit of an IC hospital located in Vic (Catalonia) between April 2022 and July 2024 who met at least one of the following conditions, were included: i) new identification of CCP or ACP on admission; ii) a change in identification with respect to previous CCP or ACP status; iii) not having an ICP or needing it to be updated. The exclusion criteria were: patients who had already been identified and had an ICP that required no changes on discharge from the unit; inability to provide informed consent; family member declining participation; expected length of hospital stay <72 h, as it was considered that this timeframe did not consistently allow sufficient time to conduct interviews with the patient and/or caregiver, nor to undertake a SDM process that took into account the patient’s values and preferences and enabled the development of an ICP, which constituted a core component of the intervention; and critically ill patients.

### Assessment of the implementation of the coordinated care model

A situational diagnosis based on a comprehensive geriatric assessment was made during the admission process and the patient’s needs were identified. Patients who met the criteria for CCP or ACP were identified by consensus of the interdisciplinary care team for the patients (geriatrician, nurse, and social worker) [21], and an ICP was developed and agreed upon with the patient/family in a SDM process [13]. The ICP included the main care goal, specific goals, and the preferred place of care for any future intercurrent illness.

The co-ordination model between IC and PC was based on the application of two sequential strategies:

First strategy (the first 63 patients): the proposed ICP was incorporated into the IC discharge summary, with the patient identified as having a CCP or ACP, the main care goal, specific goals and place of care for any new intercurrent problems.

Second strategy (the remaining 97 patients): in addition to the changes to the discharge summary, an electronic alert mechanism was implemented using the routine pre-discharge workflow, a standardized electronic record within the Catalan healthcare system aimed at facilitating continuity of care with PC. In the context of the study, the responsible physician completed the free text field of this record on the day of hospital discharge, reporting the identification as CCP or ACP and the ICP developed in IC. This information was automatically received by the patient’s PC team. Although this mechanism was already used routinely, the content of the information transmitted constituted a new component of the intervention; therefore, no formal training was provided, although PC teams were informed about the use of this channel.

Implementation of the model was considered successful if, at 3 months after discharge, the patient was identified as having a CCP or ACP by PC and the ICP was updated in the SCR, and accessible to all healthcare providers. This verifications was performed through a passive review of the record by the research team.

### Assessment of the effectiveness of the coordinated care model

The use of healthcare resources by patients was analysed for the 6 months preceding hospitalisation and for 6 months after discharge. Use of healthcare services was assessed by recording the number of contacts with PC, the number of admissions to the acute care hospital (ACH) and the two IC hospitals in the area, and visits to the emergency department (ED). Data were collected from SCRs.

### Measurements

Data were collected on the following variables:

Demographic variables: age, sex and place of residence.

Clinical variables: the following were collected at baseline: i) reason for hospitalisation (intercurrent medical condition –defined as any acute medical problem or decompensation of a chronic disease, behavioural disorder, hip fracture, other surgical complications, geriatric syndromes –falls, delirium, polypharmacy, pressure ulcers, or dysphagia-); ii) total number of morbidities, designated as chronic conditions included in the Johns Hopkins ACG System’s Expanded Diagnostic Clusters [22]; as “other morbidities”, patients were considered to have a depressive disorder if noted in the patient records or if they took specific pharmacological treatments; iii) stage of dementia as determined by the Global Deterioration Scale (GDS) [23].

Functional variables: the Barthel Index (BI) was used to assess competency in basic activities of daily living at baseline and on discharge [24].

Frailty Index: measured on admission and on discharge using the VIG-Frail frailty index [25,26]. VIG-Frail scores were classified as: i) VIG-Frail <0.20: no frailty; ii) VIG-Frail 0.20–0.35: mild frailty; iii) VIG-Frail 0.36–0.50: moderate frailty; iv) VIG-Frail >0.50: severe frailty.

Overall goals of care: The proposed patient main care goals were prolonging survival, maintaining function or prioritising symptom control. These were established based on a situational diagnosis, frailty and the preferences of the patient and agreed upon with the caregiver [27].

This study was approved by the Ethics Committee of the Consorcio Hospitalario de Vic (Vic Hospital Consortium) on 28/09/2021 (CEI (Research Ethics Committee) code number 2021175 and own code PR 306). As it involved patients affected by dementia, informed consent was provided by the primary carer.

### Sample size

Accepting an alpha risk of 0.05 and a statistical power greater than 0.8 in a bilateral contrast, 156 subjects were needed to detect a significant difference between the initial percentage of 24% of patients with an ICP to 40% with ICPs at 3 months from discharge and after implementation of the coordinated care model between IC and PC. It was estimated that the loss to follow-up rate would be 20%.

### Statistical analysis

The statistical analysis was performed with the IBM SPSS version 29.0 statistical software package. The results of the categorical variables were expressed as absolute and relative frequencies, and those of the continuous variables were analysed using both parametric and non-parametric statistical tests, depending on the level and distribution of the data (as mean and standard deviation (SD) or median, first quartile (Q1) and third quartile (Q3) and minimum and maximum values). The Chi-Square test or Fisher’s exact test in 2 × 2 tables (where the expected frequencies were lower than 5) were used for categorical variables. Statistical tests for paired data were used to analyze the impact of the intervention: the McNemar test for categorical variables; Student’s paired-sample t-test for normally distributed quantitative variables; and the Wilcoxon Test for quantitative variables that were not normally distributed. A p-value less than 0.05 was considered to be statistically significant.

## Results

Of the 160 patients included in this study, 56.3% (n = 90) were women and the mean age was 84.9 ± 7.0 years. Overall, the patients included had moderate dependency for daily living activities, with a mean Barthel Activities of Daily Living Index score of 59.3 ± 25.8, and moderate frailty, with a mean VIG-Frail score of 0.42 ± 0.1. Patients with severe frailty accounted for 30% (n = 48) of the sample. The demographic, clinical, functional and cognitive characteristics at baseline, according to provenance and reason for admission, are described in Table 1.

**Table 1:** Baseline data.


		TOTAL n = 160

Age, mean (SD)		84.95 (7.0)

Sex, n (%)	Women, n (%)	90 (56.3%)

	Men, n (%)	70 (43.8%)

Origin, n (%)	Home, n (%)	145 (90.6%)

	Nursing home, n (%)	15 (9.4%)

Barthel index (BI), mean (SD)		59.34 (25.8)

Frailty index (VIG-Frail), mean (SD)		0.42 (0.1)

Cognitive status, n (%)	Mild dementia (GDS 4), n (%)	8 (5.0%)

	Moderate dementia (GDS 5–6C), n (%)	90 (56.3%)

	Advanced dementia (GDS > 6C), n (%)	62 (38.8%)

Mean comorbidity, mean (SD)		6.04 (2.2)

Previously identified as a patient with a complex or advanced chronic condition, n (%)	CCP, n (%)	64 (40.0%)

	ACP, n (%)	13 (8.1%)

	No, n (%)	83 (51.9%)

Existing ICP	Yes, n (%)	22 (28.6%)

	No, n (%)	55 (71.4%)

Patient’s provenance, n (%)	Acute care hospital, n (%)	81 (54%)

	A&E/Observation, n (%)	50 (33.3%)

	Place of residence, n (%)	19 (12.7%)

Reason for hospitalisation	Intercurrent medical condition, n (%)	83 (55.3%)

	Behavioural disorder, n (%)	18 (12%)

	Femur fracture, n (%)	25 (16.7%)

	Geriatric syndromes, n (%)	24 (16%)


Abbreviations: SD: Standard deviation, GDS: Global Deterioration Scale, CCN: patients with complex chronic care needs, ACD-LLE: patients with advanced chronic diseases and limited life expectancy, ICP: individualised care plan.

On discharge, 58.8% (n = 94) were identified as having CCP, and 41.3% (n = 66) as having ACD-LLE. In 63.1% (n = 101) of cases, the goal established by consensus with patients/families was maintaining function, prioritizing symptom control in 35.6% (n = 57), and prolonging survival in 1.3% (n = 2). On discharge, the mean BI score was 47.4 ± 25.0, and the mean VIG-Frail score was 0.49 ± 0.10. Twenty six per cent (n = 40) of patients were discharged to a new care home setting.

**Figure 1:**
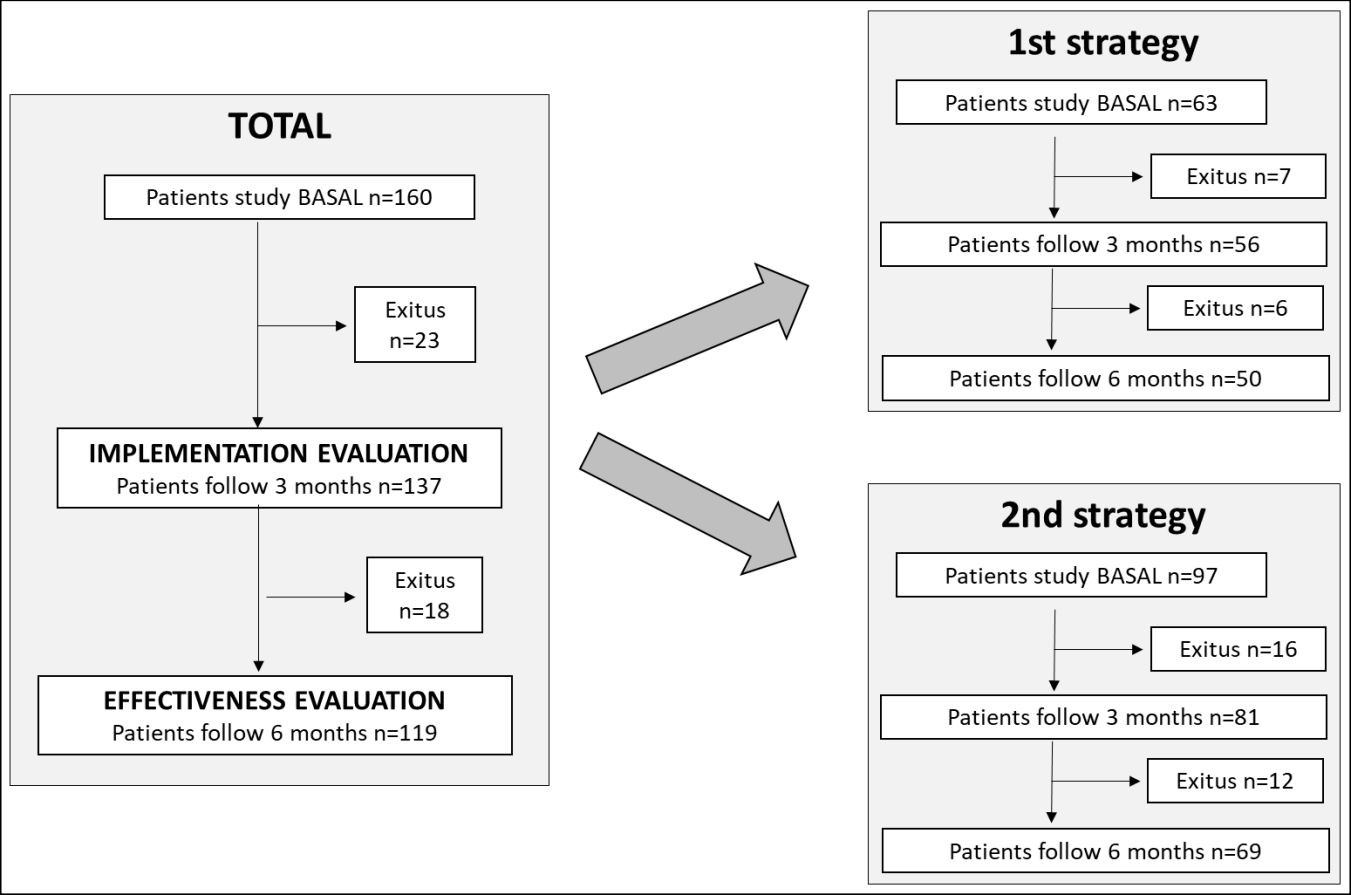
Flow diagram.

At 3 months of discharge, 14.4% (n = 23/160) of the patients had died (Figure 1).

Of the 137 patients who were still alive, 77.4% (n = 106/137) were identified as having CCP or ACP in the SCR. Of these, 46.0% (n = 63/137) had already been identified pre-hospitalisation, representing a 31.4% increase in recorded identification in their medical record (p < 0.001). When analysed by the strategy applied, we found that with the first strategy, of the 56 patients still alive at 3 months, 71.4% (n = 40/56) had been identified, of whom 46.4% (n = 26/56) were already identified pre-hospitalisation, representing a 25.0% increase (p < 0.001). With the second strategy, of the 81 patients who were still alive, 81.5% (n = 66/81) were identified, and of these, 45.7% (n = 37/81) had been identified previously, representing an increase of 35.8% (p < 0.001).

At 3 months of discharge, of patients who were alive and identified (n = 106) 41.5% (n = 44/106) had an ICP recorded in the SCR. Of these, 17.9% (n = 19/106) already had a ICP pre-hospitalisation, which means there was a 23.6% increase (p < 0.001). When analysed according to the strategy applied, we found that with the first strategy 35% (n = 14/40) of patients who were identified as having CCP or ACP had an ICP in the record at 3 months, 22.5% (n = 9/40) of whom already had one pre-hospitalisation, representing an increase of 12.5% (p = 0.126). With the second strategy, there was an ICP recorded for 45.5% (n = 30/66), of whom 15.2% (n = 10/66) had a previous one, representing an increase of 30.3% (p < 0.001).

With respect to the use of healthcare resources, we found that in the 6 months pre-hospitalisation, 15% (n = 24) of patients had required admission to an IC unit, 17.5% (n = 28) to an AC hospital, and 48.8% (n = 78) made at least one visit to ED. The mean number of visits to PC services was 10.38 (SD 9.4), with a median of 8 (Q1:3-Q3:14). No statistically significant differences were observed in the use of resources in the 6 months pre-hospitalisation between patients who had died by 6 months and those who survived.

At 6 months of discharge, 25.6% (n = 41) of patients had died. Within 6 months of discharge, 15.1% (n = 18) of patients required admission to an IC unit, 15.1% (n = 18) to an AC hospital, and 34.5% (n = 41) made at least one visit to the ED. The mean number of visits to PC services was 11.03 (SD 10.4), with a median of 8 (Q1:5-Q3:15).

**Figure 2:**
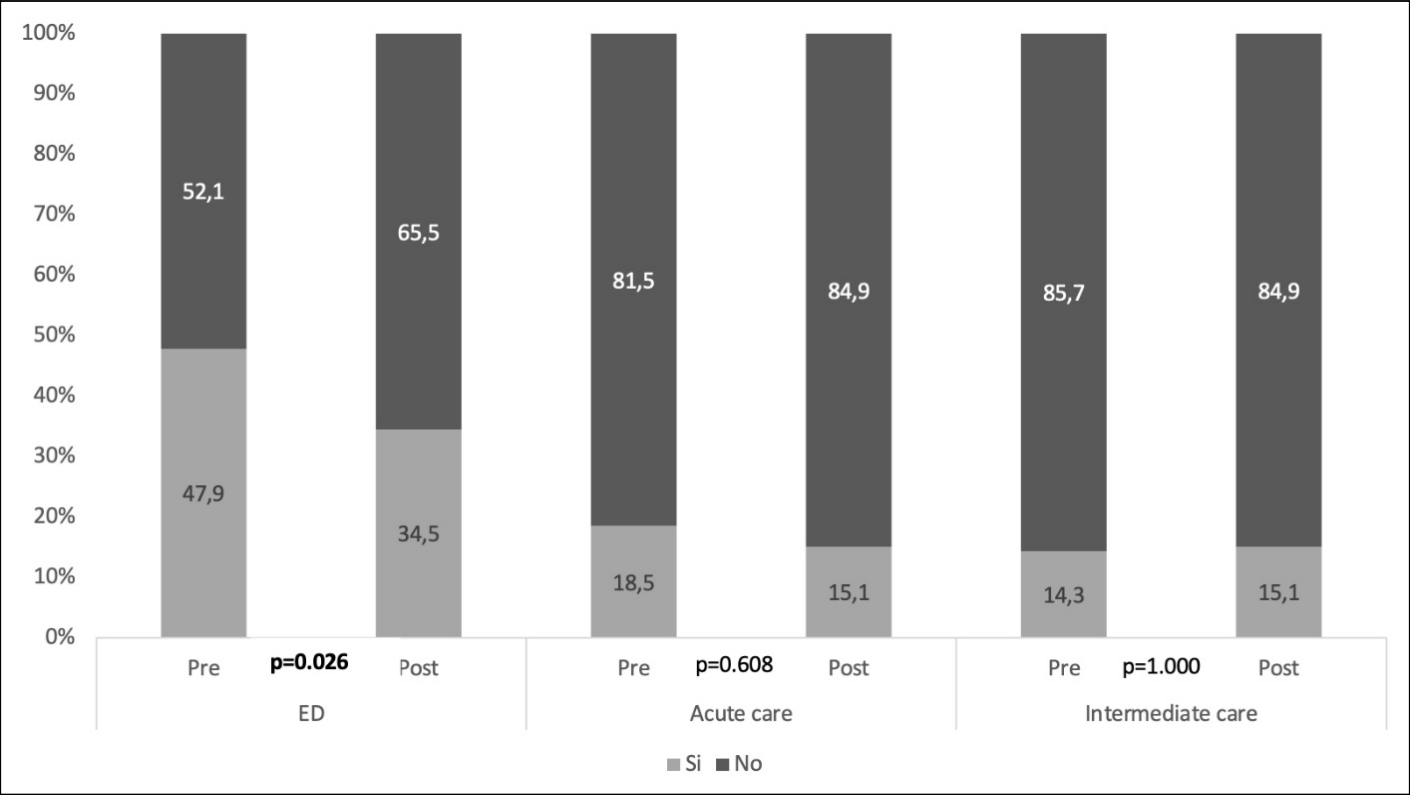
Use of resources pre and post hospitalisation in an IC hospital.

Overall, at 6 months post-discharge, we found a 13.4% decrease in ED use. Of the 119 patients still alive at 6 months, we compared resource use between the 6 months pre-hospitalisation and 6 months after discharge. We found a statistically significant decrease in the use of ED, 54.4% (n = 31) of the patients who had visited the ED in the 6 months prior to hospitalisation did not visit ED in the 6 months (p = 0.026) post-discharge, 24.2% (n = 15) of the patients who had not visited ED in the 6 months pre-hospitalisation, visited it in the 6 months post-discharge, with no statistically significant differences observed in the use of other resources (Table 2 and Figure 2).

**Table 2:** Use of resources pre and post hospitalisation an IC hospital.


USE OF HOSPITAL CARE RESOURCES			

VISITS to ED(6 months pre-hospitalisation)	VISITS to ED (6 months post-discharge)		

	NO	YES	p

**NO**	47 (75.8%)	15 (24.2%)	**0.026**

**YES**	31 (54.4%)	26 (45.6%)	

**Intermediate care hospital** **(6 months pre-hospitalisation)**	**Intermediate care hospital (6 months post-discharge)**		

	**NO**	**YES**	**p**

**NO**	84 (82.4%)	18 (17.6%)	1.000

**YES**	17 (100.0%)	0 (0.0%)	

**Acute care hospital** **(6 months pre-hospitalisation)**	**Acute care hospital (6 months post-discharge)**		

	**NO**	**YES**	**p**

**NO**	82 (84.5%)	15 (15.5%)	0.608

**YES**	19 (86.4%)	3 (13.6%)	

**USE OF PRIMARY CARE RESOURCES**			

**Visits to Primary Care services**	**6 months pre-hospitalisation**	**6 months post-discharge**	

**Median (Q1-Q3)**	8 (3.0; 14.0)	8 (5.0; 15.0)	0.536


Pre–post comparisons were performed using McNemar’s test. ED: emergency department.

## Discussion

A model for care coordination between IC and PC, geared towards patients with dementia and complex or advanced chronic conditions and based on the proactive identification of these patients, the development of an ICP through in-depth interviews within a SDM process together with the patient/family, and the exchange of structured information between different levels of care through the discharge summary and computer alerts, has supported the increase in record-keeping in the SCR, in the identification of patients with complex or advanced chronic conditions and the drafting of their respective ICPs by the PC services in our area. In addition, the increase in the availability of information in the SCR may have contributed to greater continuity between care levels and, hence, to a decrease in the use of ED by these patients, which suggests a possible impact in terms of effectiveness.

To meet the needs of patients with CCP and ACP, patient-centred coordinated care models are needed between care levels [28]. In this study, we assess a model of care coordination between IC and PC for patients with dementia, multimorbidity and frailty who are identified as having CCP or ACP during their admission to an IC hospital, in regard to implementation of the model and its effectiveness. This study contributes to the existing literature by providing real-word clinical practice evidence of integrated care principles for people with frailty, positioning IC as a key coordination hub with PC and focusing on a particularly vulnerable population, namely people with dementia [29].

The first practical consequence of the study, which is related to implementation of the care model, is seen by the increase in the recording of patients identified as having a CCP or ACP and their ICPs by PC in the 3 months post-discharge. Although a direct causal relationship cannot be established, this improvement coincides temporally with the incorporation of two key elements in the coordination process: the newly structured discharge summary and the sending of a computer alert to the PC teams. Both elements, the structuring of clinical information and the technological support necessary for its effective exchange between levels of care, have been identified in the literature as fundamental components of integrated care [30]. Other facilitators may also have contributed, such as the existence of an already established relationship of trust and respect between the PC and IC teams [31,32]. Nonetheless, possible hurdles that may have influenced the absence of validation or recording of information by some PC teams should also be considered. Among these, the high care burden that makes it difficult to find the time needed, high turnover of healthcare practitioners that implies less experience or knowledge in the fields of complex and/or advanced chronic conditions, and the fact that integrated work has sometimes been perceived to be a source of additional effort rather than as a more effective way of working [8,32,33].

The second consequence, related to the effectiveness of the implementation of the care cooperation model in the area, was seen in the use of health resources. Our study showed a lower use of ED in the six months post-discharge, with no statistically significant differences observed in the use of other healthcare resources. In this context, the absence of changes in the number of primary care visits is interpreted as appropriate and planned follow-up of patients, consistent with the objectives of the model to improve continuity of care and reduce the use of unplanned resources.

There are a wide variety of integrated care strategies with the aim of reducing the use of healthcare resources, but findings are still insufficient to draw any conclusions regarding significant benefits in the use of hospital resources [34,35]. Still, some coordinated care models that focus on patients with complex profiles have shown significant reductions in unplanned care and hospitalisations. The Gundersen Health [36], which focuses on high-risk patients, achieved a 64% cumulative reduction in expenditures arising from ED visits and unscheduled hospitalisations over 24 months, as well as a decrease of more than 50% in the number of hospitalisations. These results reinforce the idea that well-structured interventions can improve care efficiency, especially when they are maintained over the medium term. Indeed, the authors underline that the most notable benefits of the program became apparent after at least two years of implementation, suggesting that the duration of the intervention is a determining factor in the impact it has. Much of the evaluation literature suggests that integrated care is unlikely to deliver changes to activities and costs in the short term. Hence, integrated care should be setting broader objectives about population health and patient and staff experience [37]. We need new metrics and a profound transformation to allows us to move from a system that values volume of activity and processes to an integrated care model focused on overall outcomes and on what has value for the patient [38,39]. To achieve this objective requires not only structural reorganization, but also the development of collective capacities that favour continuous learning, interdisciplinary cooperation and effective implementation of knowledge in clinical practice. In this sense, communities of clinical practice appear as very useful complementary instruments. Their ability to facilitate the translation of evidence into concrete actions, as well as to promote continuous improvement through peer-to-peer interaction, makes them a strategic component towards achieving value-based integrated care. Integrating these approaches can not only improve clinical outcomes and the patient’s experience, but can also contribute to the sustainability of the health system in the medium to long term [40,41].

We also want to highlight the key role that IC plays in the healthcare system. IC is an essential component of integrated care, providing interventions aimed at preventing in-hospital complications, optimising functional recovery processes and ensuring continuity of care, especially between different levels of care and care settings [35]. In our study, 46% of the patients were admitted from ED/Observation or their place of residence (STEP-UP model), which, in line with other published reports, reinforces the role of IC in the prevention of avoidable hospitalisations and in the improvement in ED performance [42]. IC provides a multidisciplinary care model that is highly specialized in geriatrics and palliative care, geared towards the needs of patients with complex or advanced chronic conditions. Its importance lies not only in the professional experience and the integrated approach of the team, but also in the duration of hospitalisation, which allows carrying out a comprehensive geriatric assessment and to plan post-discharge care.

Our coordination model is highly transferable, and its key components are potentially applicable to other settings. Its implementation would require the availability of IC or equivalent services, functional coordination mechanisms based on the structured transfer of clinical information aimed at continuity of care, as well as shared information systems, with the necessary organisational adaptations according to the local context.

## Limitations, Strengths and Future Work

This study has several limitations that should be considered when interpreting the results. In the first place, the pre-post quasi-experimental study design with no control group limits the possibility of establishing causal relationships. The changes seen during the implementation of the care model and in the use of healthcare resources may have been influenced by external factors not directly related to the intervention, such as the natural clinical course. However, it should be noted that the study was conducted as part of routine clinical practice at the hospital, which justifies the choice of design. The inclusion of a control group would have meant depriving some patients of a coordinated care model that is part of standard care practice. Secondly, the results of the implementation of the care model (at 3 months) and those assessing its effectiveness (at 6 months) were analysed only for patients still alive at the time of the analyses. This may have led to a survivorship bias, as it excludes the deceased patients, who may have had different patterns of use of resources or response to the intervention. However, we analysed the prior use of resources by both the patients who were deceased at 6 months and the survivors and observed no significant differences.

It should also be considered that we have not assessed whether the absence of validation or recording of information by some in our study is due to discrepancies between IC and PC concerning the identification of CCP or ACP and the proposed ICP.

This study has several strengths. First, it presents a practical, real-life implementation of a conceptual model for an especially vulnerable population: patients with dementia and highly complex conditions. This makes the intervention an example of translational research, as it contributes to narrowing the gap between healthcare theory and practice. Secondly, it institutes an innovative strategy using a coordinated care model that combines technological and clinical factors: structuring information in the discharge summary with specific ICP proposals, sending computer alerts to the PC teams and more active use of the SCR, not only as an information repository, but as a dynamic coordination tool. The study also addresses significant gaps in knowledge around coordination between levels of care, providing preliminary results on effectiveness and presenting an organizational model with potential for scalability to other healthcare system settings. Finally, it integrates key aspects for current healthcare system transformation processes, such as digitalisation, efficient use of clinical information, and the promotion of person centred care.

In future studies, essentially for cases that are more complex, we plan to incorporate more structured methodologies, similar to case conferences, that would allow the healthcare practitioners involved in the care of the patient to agree on a single individualised plan to be shared between them, the person, and their care setting [43]. In addition, the use of qualitative approaches, such as interviews or surveys with professionals, could help explore barriers, facilitators, and potential divergences in the identification and validation of ICP across care levels. A possible avenue of future research can be to assess whether the care received in cases of decompensation is in accordance with the values, preferences and main care goal agreed upon with the patients, considering that it is the priority outcome and therefore of utmost importance in the ICPs [44]. In patients with MACA, future studies should also evaluate end-of-life care quality and family or caregiver experience as key outcomes.

## Conclusion

Patients with the most complex health profiles are major consumers of healthcare resources, yet they often receive fragmented and suboptimal care, in part because of a lack of communication and coordination among healthcare professionals [36]. Attention to the needs of persons and their social circle should be one of the objectives of the healthcare response by care teams and organizations. Persons with complex or advanced chronic conditions require comprehensive, integrated responses that are appropriate according to their stage of life. The implementation of a coordinated care model between IC and PC in our area has helped improve the identification of people with complex or advanced chronic conditions, the recording of their ICPs, and reduce their use of ED. This model has relevant practical implications and may be scalable to other regions with shared information systems and intermediate care services, subject to appropriate organisational adaptations.

## Patient and Public Involvement

Patients or people with lived experience were not involved in the design, conduct, reporting or dissemination of this research.
